# Easy One-Pot Low-Temperature Synthesized Ag-ZnO Nanoparticles and Their Activity Against Clinical Isolates of Methicillin-Resistant *Staphylococcus aureus*

**DOI:** 10.3389/fbioe.2020.00216

**Published:** 2020-03-19

**Authors:** Atanu Naskar, Sohee Lee, Kwang-sun Kim

**Affiliations:** Laboratory of RNA Biochemistry & Superbacteria Research, Department of Chemistry and Chemistry Institute for Functional Materials, Pusan National University, Busan, South Korea

**Keywords:** low-temperature solution synthesis, Ag-ZnO nanoparticles, antibacterial activity, Gram-positive bacteria, MRSA

## Abstract

Antimicrobial resistance (AMR) is widely acknowledged as a global health problem, yet the available solutions to this problem are limited. Nanomaterials can be used as potential nanoweapons to fight against this problem. In this study, we report an easy one-pot low-temperature synthesis of Ag-ZnO nanoparticles (AZO NPs) and their targeted antibacterial activity against methicillin-resistant *Staphylococcus aureus* (MRSA) strains. The physical properties of the samples were characterized by X-ray diffractometry (XRD), transmission electron microscopy (TEM), and X-ray photoelectron spectroscopy (XPS). Furthermore, minimum inhibitory concentration (MIC), zone of inhibition (ZOI), and scanning electron microscopy (SEM) images for morphological characterization of bacteria were assessed to evaluate the antibacterial activity of AZO NPs against both Gram-negative [*Escherichia coli* (*E. coli*) and *Acinetobacter baumannii* (*A. baumannii*) standard and AMR strains] and Gram-positive (*S. aureus*, MRSA3, and MRSA6) bacteria. The AZO NPs showed comparatively better antibacterial activity against *S. aureus* and MRSA strains than Gram-negative bacterial strains. This cost-effective and simple synthesis strategy can be used for the development of other metal oxide nanoparticles, and the synthesized nanomaterials can be potentially used to fight against MRSA.

## Introduction

Antimicrobial resistance (AMR) is the ability of a given microbe to resist the effects of multiple antibiotics (Huijbers et al., 2015; Prestinaci et al., 2015). AMR is easily recognized in hard-to-treat pathogens and has become an alarming issue complicating health care and many other sectors (Eliopoulos et al., 2003; Jasovsky et al., 2016). For instance, methicillin-resistant *Staphylococcus aureus* (MRSA) is one of the most well-known AMR bacterial species for which immediate intervention is necessary, but even the long considered last-resort antibiotic vancomycin cannot be used in the treatment of MRSA infections since vancomycin-resistant *S. aureus* (VRSA) strains have emerged (Naskar and Kim, 2019; Naskar et al., 2020). In addition, AMR *S. aureus* species are one of 12 families of priority pathogenic bacteria listed by the World Health Organization (WHO) for which antibiotics are urgently needed (World Health Organization [WHO], 2017). Several new currently approved oxazolidinone class antibiotics, including Sivextro (Hall et al., 2018), tigecycline (Hall et al., 2018), and LCB01-0371 (Jeong et al., 2010) to eradicate *S. aureus* species resistant to last-resort antibiotics have been developed. However, it is possible that bacteria might continue to evolve to evade this new class of last-resort antibiotics, and it takes much time to find other alternatives and their mechanism of action in response to a newly generated resistant strain. Therefore, new alternatives to antibiotics are desperately needed for the fight against AMR pathogens.

In this present scenario, nanomaterials have emerged as both viable and versatile alternatives to current antibiotics to fight against AMR bacteria as it showed effectiveness in low dosages also where chances of bacteria getting resistance is also less (Regí et al., 2019). The main advantage of nanoparticles as antibacterial agents (i.e., nanoweapons) is that they function via a multiple target approach compared to the single target approach of antibiotics to inhibit the growth of bacteria (Naskar et al., 2016; Baptista et al., 2018). Therefore, it is harder for bacteria to attain resistance toward nanoparticles. A large surface area to volume ratio is also one of the major advantages of nanoparticles for their use in various biomedical applications including antibacterial activity (Navya and Daima, 2016; Naskar et al., 2018). Among such nanomaterials, metal- and metal oxide-based nanoparticles have been preferred by researchers to combat AMR bacterial cells (Wang et al., 2017). However, silver (Ag) nanoparticles (NPs) have been the most effective and promising antibacterial candidates since ancient times due to their inhibitory and antibacterial properties against microorganisms, including 16 major species of bacteria (Lee and Jun, 2019). Moreover, zinc oxide (ZnO) NPs are another well-known antibacterial nanomaterial (Sirelkhatim et al., 2015; Hassan et al., 2017; Kumar et al., 2017; Naskar et al., 2017). ZnO nanoparticles have been recognized as a safe material by the US Food and Drug Administration [(21CFR182.8991) (Food and Drug Administration (FDA), 2015)]. Therefore, Ag-ZnO (AZO) NPs can be a potential alternative to conventional antibiotics in the fight against AMR bacteria.

Several methods like sol–gel (Lu et al., 2011) hydrothermal (Zhang and Mu, 2007) co-precipitation (Md Subhan et al., 2014), and plasma-assisted chemical vapor deposition (Simon et al., 2011) have been successfully reported for the synthesis of AZO NPs. However, all of these processes use high temperature and high pressure with long reaction times and multiple steps, which limit the use of AZO NPs in various applications (Matai et al., 2014). Very few reports, in fact, are available regarding the single step and low temperature synthesis of AZO NPs for the killing of AMR pathogens (especially MRSA pathogens) despite the immense potential for AZO NPs as antibacterial agents.

In the present work, a simple one-pot low-temperature synthesis method was developed to successfully synthesize AZO NPs from simple metal precursors and hydrazine hydrate. The antibacterial activity of the synthesized nanoparticles was evaluated for AMR strains of Gram-positive bacteria, including MRSA strains, and Gram-negative bacteria.

## Materials and Methods

### Synthesis of ZnO (ZO) and Ag-ZnO (AZO) NPs

Initially, a fixed quantity (1 g) of zinc nitrate hexahydrate (Zn[NO_3_]_2_⋅6H_2_O, Merck) and requisite amount of silver nitrate (AgNO_3_, ACS, ≥99.9%) [0 and 5 atomic percent (at%) with respect to Zn] was uniformly dispersed in 50 mL of deionized water (DW) with continuous stirring for 60 min at room temperature. In the next step, 1 mL of hydrazine hydrate (N_2_H_4_⋅H_2_O, Merck, 99–100%) was added dropwise to the reaction mixture with continuous stirring. Subsequently, the mixture was ultrasonicated for 10 min in a water bath ultrasonicator. Now, gray colored precipitation was clearly visible in the reaction beaker. The same steps, i.e., dropwise addition of hydrazine hydrate and ultrasonication, were repeated until the pH of the medium reached eight. Afterward, the precipitate of solid materials was separated by centrifugation and DW and ethanol were used for washing. Finally, the samples were dried in an oven at ∼60°C for 24 h. The products were designated as ZO and AZO where the at% used in the precursors was 0 and 5, respectively.

### Characterization

#### Material Properties

X-ray diffraction (XRD) using an X-ray diffractometer (D8 Advance with DAVINCI design XRD unit, Bruker) with nickel filtered Cu Kα radiation source (λ = 1.5406 Å) was used to evaluate the structures of ZO and AZO. The diffraction patterns were collected in the 2θ range of 20–80°. Moreover, the microstructure of the representative sample of AZO was assessed by transmission electron microscopy (TEM; Bruker Nano GmbH). Carbon coated 300 mesh Cu grids were used for placing the samples. An Axis Supra Scanning X-ray photoelectron spectroscopy (XPS) microprobe surface analysis system was used to assess a representative sample of AZO by scanning the binding energy ranging from 200 to 1,200 eV to determine the chemical state of elements. The C 1s peak position at 284.5 eV was used as the binding energy reference.

#### Growth of Bacteria for Evaluation of the Antibacterial Activity

Generally, antibacterial activity was evaluated according to a previous report (Naskar et al., 2020) using BBL^TM^ Mueller-Hinton Broth (MHB, Becton Dickinson) grown bacterial strains including *E. coli* (ATCC 25922), *A. baumannii* (ATCC 19606), *S. aureus* (ATCC 25923); AMR strains of *E. coli* (1368), *A. baumannii* (12001); and different MRSA clinical isolates (Shin et al., 2019). Briefly, the MHB medium was used for the inoculation of single colonies of bacteria, which were incubated at 37°C overnight, followed by dilution of the cells to an optical density of 0.5 McFarland turbidity standard using Sensititre^TM^ Nephelometer (Thermo Scientific). The cell cultures were used within 30 min after dilution to prepare samples for minimum inhibitory concentration (MIC) assay (section “MIC for Evaluation of the Antibacterial Activity”) or scanning electron microscopy (SEM) analysis (section “Morphological Characterization of Bacteria”) to assess the antibacterial activity of NPs (ZO and AZO) and characterize cell morphology, respectively.

#### MIC for Evaluation of the Antibacterial Activity

All bacteria were incubated overnight in the MHB medium. The number of cells was determined with a Sensititre^TM^ Nephelometer to a 0.5 McFarland standard and diluted at a ratio of 1/1,000 in MHB. The ZO and AZO samples (5 mg/mL each) were prepared by serial dilution with DW to obtain concentrations from 250 to 10 μg/mL. Then, 90 μL of the targeted bacterial medium was inoculated with 10 μL of each diluted sample. The bacterial cells were incubated by shaking at 500 rpm for 16 h at 37°C. The MIC was evaluated after this process.

#### Agar Well Diffusion Method for Evaluation of the Antibacterial Activity

The antibacterial activity of ZO and AZO against the bacterial strains of *E. coli*, *A. baumannii*, *S. aureus*, MRSA3, and MRSA6 was further evaluated with the agar well diffusion method. First, 500 μL of cultured bacterial cells were mixed with 25 mL of MHB-agar, poured into sterile petri dishes (ϕ = 90 mm), and solidified. Then, five holes, 6 mm in diameter each, were aseptically punched through the surface with a sterile plastic rod. Afterward, 20 μL of ZO or AZO (5 mg/mL), polymyxin B or kanamycin (5 mg/mL, Sigma-Aldrich), or DW was added for the experimental group, the positive control for Gram-negative or -positive strains, and the negative control group respectively. The plates were then incubated for 24 h at 37°C. Finally, the antibacterial activities were evaluated by measuring the diameter of the zone of inhibition (ZOI) around the wells using a ruler.

#### Morphological Characterization of Bacteria

At first, prepared bacterial cells through the same process as described in section “Growth of Bacteria for Evaluation of the Antibacterial Activity” were diluted at a ratio of 1/1,000 in the MHB medium according to MIC assay. 900 μL of prepared cells were incubated with 100 μL of the three final concentration 0, 10, and 20 μg/mL of AZO for 16 h at 37°C with vigorous shaking. After that, the incubated cells were harvested by centrifugation at 12,000 rpm for 1 min to get a pellet. Then this pellet was resuspended in 500 μL of phosphate buffered solution (pH 7) containing 2% formaldehyde and 1% glutaraldehyde, and centrifuged again. Subsequently, the obtained cell pellet was washed twice with DW and resuspended in 1 mL of DW for further experimentation. A 5 μL aliquot was taken from the suspension and deposited on a silicon wafer (5 mm × 5 mm in size, Namkang Hi-Tech Co., Ltd.) to dry at room temperature. Finally, the air-dried wafer was subjected to SEM analysis using VEGA3 (TESCAN), a versatile tungsten thermionic emission SEM system, according to the manufacturer’s protocol.

## Results and Discussion

### Material Properties

#### Phase Structure

**FIGURE 1:**
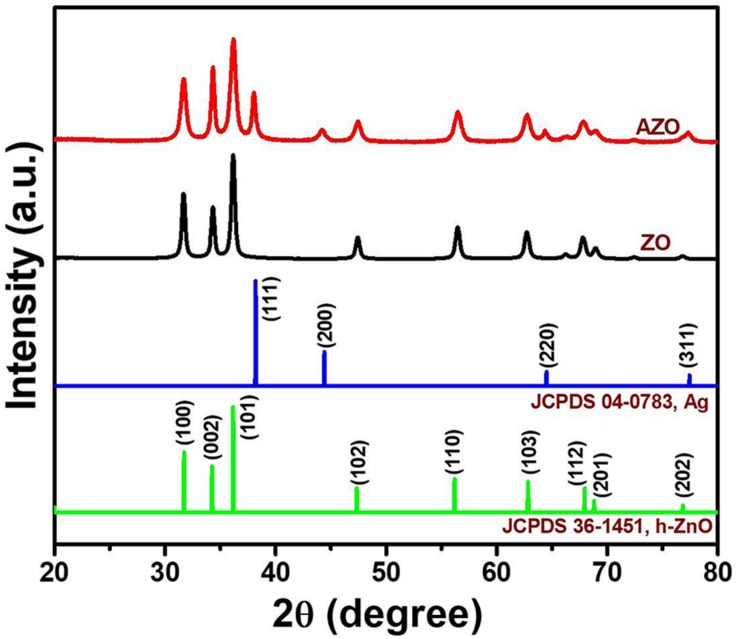
XRD patterns of ZO and AZO samples.

XRD was used to analyze the crystalline phase of samples. Figure 1 shows the XRD patterns of as-synthesized ZO and AZO samples. The obtained XRD patterns of the samples were consistent with hexagonal ZnO (h-ZnO) (JCPDS 36-1451) (Saloga and Thünemann, 2019). Moreover, some additional peaks can be seen at ∼38.1°, ∼44.3°, ∼64.5°, and ∼77.4° for AZO samples, which corresponded to the crystal planes of cubic Ag (JCPDS 04–0783) along (111), (200), (220), and (311), respectively (Nogueira et al., 2014). Therefore, the formation of AZO NPs was successfully confirmed.

#### Morphology and Microstructure

**FIGURE 2:**
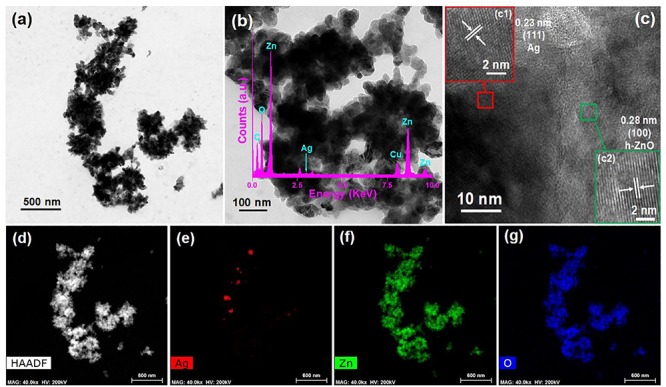
TEM image **(a,b)** and HRTEM image **(c)** of AZO sample where **(c1)** and **(c2)** show the HRTEM images of the particles of Ag and ZnO, respectively with (**b** inset) TEM-EDS spectrum, **(d)** HAADF image, and elemental mappings of **(e)** Ag, **(f)** Zn, and **(g)** O.

Transmission electron microscopy (TEM) was conducted systematically to further investigate the formation of ZO/AZO nanoparticles. The TEM image of the AZO sample and the corresponding HRTEM and HAADF images are shown in Figures 2a–d, respectively. The HRTEM image (Figure 2c) of the AZO sample shows distinct lattice fringes with an interplanar distance of 0.28 nm, corresponding to the (100) plane of hexagonal ZnO (Ren et al., 2016). This observation confirmed the presence of hexagonal ZnO in the AZO sample. Moreover, HRTEM showed lattice fringes having an interplanar distance of 0.23 nm (Figure 2c), which can be matched with (111) of Ag NP (Sareen et al., 2015). Therefore, the TEM characterization of the microstructure of the AZO sample confirmed the presence of both nanoparticles of ZnO and Ag, which corroborated with the XRD result (Figure 1). Additionally, the TEM with energy-dispersive X-ray (TEM-EDX) spectral analysis of the AZO sample confirms the presence of Zn and O and corroborates that ZnO NPs were formed (Figure 2b). The presence of Ag suggests the formation of Ag NP in the Azo sample. The presence of C and Cu in the TEM-EDX spectrum can be attributed to the carbon coated Cu grid used for the TEM measurements. The elemental mapping result of Ag (Figure 2e), Zn (Figure 2f) and O (Figure 2g) for the representative AZO sample reveals the distribution Ag, Zn, and Au elements in the sample.

#### XPS Spectra

**FIGURE 3:**
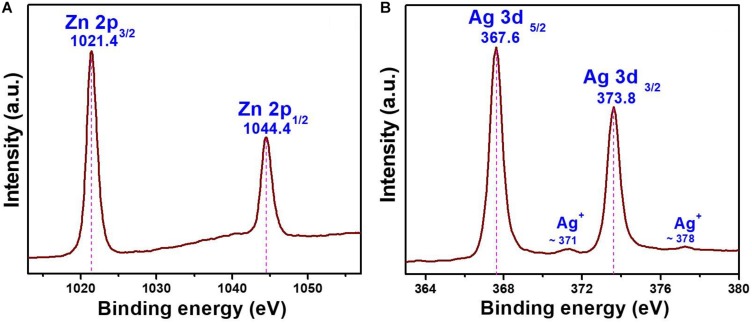
XPS binding energy spectra of AZO **(A)** Zn *2p* and **(B)** Ag *3d* core levels.

The oxidation state of the chemical elements present in the AZO sample was evaluated by XPS analysis, and the binding energy signals of the Zn *2p* and Ag *3d* core levels are shown in Figure 3. Two strong signals were observed in the binding energy signals of Zn *2p* at 1021.4 and 1044.4 eV (Figure 3A), which can be assigned to the binding energies of Zn *2p_3__/__2_* and Zn *2p_1__/__2_*, respectively (Jiamprasertboon et al., 2019). The presence of zinc as Zn^2+^ in the nanomaterial was also confirmed by the energy difference calculated between Zn *2p_3__/__2_* and Zn *2p_1__/__2_* binding energy levels, which was ∼23.0 eV (Jiamprasertboon et al., 2019). Furthermore, the formation of Ag nanoparticles was also evaluated by the binding energy signals of Ag *3d* (Figure 3B). The binding energy signals (Figure 3B) appearing at 367.6 and 373.8 eV in the XPS curve of the AZO sample can be assigned to Ag *3d_5__/__2_* and Ag *3d_3__/__2_*, respectively (Nguyen et al., 2018). This observation confirmed the formation of Ag NPs in the AZO sample. Moreover, two low intensity signals can also be seen at ∼371 and ∼378 eV. These low intensity peaks can be attributed to a trace amount of Ag^+^ ions present in the sample (Naskar et al., 2016). Therefore, the presence of metallic silver and vey less Ag^+^ could be effectively used against bacterial cells for antibacterial activity. This material property is successfully correlated with the antibacterial activity of this sample in later section of antibacterial activity.

### Antibacterial Activity

#### MIC and ZOI

The MIC values (Table 1) of the ZO and AZO samples were measured to evaluate the antibacterial effectiveness of the samples against standard strains of bacteria (*E. coli* [ATCC25922], *A. baumannii* [ATCC19606], and *S. aureus* [ATCC25923]) and AMR strains (*E. coli* 1368, *A. baumannii* 12001, MRSA3, and MRSA6). MIC determination clearly showed that the AZO sample was comparatively more effective against Gram-positive bacteria than Gram-negative bacteria. Although the AZO sample was effective against Gram-negative bacteria, its MIC was considerably very high against generic and AMR strains (100–250 μg/mL). However, the AZO sample was much more effective against Gram-positive bacterial cells; the MIC value for *S. aureus* and its AMR strains MRSA3 and MRSA6 were in the range of 25–50 μg/mL.

**TABLE 1:** Antibacterial activity of ZnO samples.

**Bacteria cells**	**Minimum inhibitory**	
	**concentration (μg/mL)**	
	**(i) ZO**	**(ii) AZO**
**Standard strains**		
(a) *E. coli* ATCC 25922	250	100
(b) *A. baumannii* ATCC 19606	>250	250
(c) *S. aureus* ATCC 25923	50	25
**AMR strains**		
(d) *E. coli* 1368	>250	250
(e) *A. baumannii* 12001	>250	250
(f) MRSA3	100	50
(g) MRSA6	100	50

*Minimum inhibitory concentration (MIC) of ZO and AZO samples against both Gram-negative and -positive bacteria cells including their AMR strains. Data shown here is one of the representative from *n* = 3.*

**FIGURE 4:**
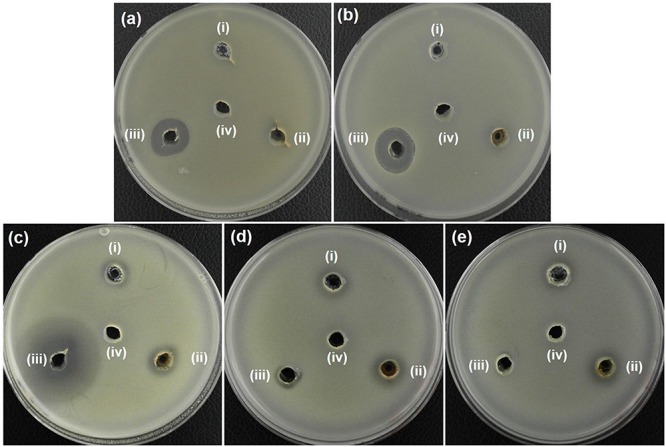
Zone of inhibition (ZOI) of ZnO samples against **(a)** *E. coli*, **(b)** *A. baumannii*, **(c)** *S. aureus*, **(d)** MRSA3, and **(e)** MRSA6. **(i)**, **(ii)**, **(iii)**, and **(iv)** represents ZO, AZO, Antibiotics, and deionized water, respectively in all the figures. Diameter of ZOI is also displayed in the Table 2 (average from *n* = 3).

In additional to the MIC determination, the agar well diffusion method was also used to further evaluate the antibacterial activity of AZO NPs. Initially, agar plates with bacterial cells were loaded with the synthesized NPs (20 μL at 5 mg/mL) and incubated for 24 h at 37°C. After that, the ZOI was measured. The bacterial growth inhibition capacity of the ZO and AZO samples against *E. coli* (Figure 4a), *A. baumannii* (Figure 4b), *S. aureus* (Figure 4c), MRSA3 (Figure 4d), and MRSA6 (Figure 4e) is provided in Table 2. The ZO and AZO NPs were unable to form an inhibition zone against Gram-negative bacterial cells of *E. coli* (Figure 4a) and *A. baumannii* (Figure 4b). These results support the MIC data (Table 1) for Gram-negative bacterial cells, from which it can be concluded that a more concentrated dispersion of AZO NPs would be necessary to obtain an inhibition zone i.e., to be effective against Gram-negative bacterial cells in the agar well diffusion antimicrobial determination. The ZOI against AMR strains (*E. coli* 1368, *A. baumannii* 12001) of Gram-negative bacterial cells was not determined, as it was assumed to be higher than the AZO sample, which was above the limit of detection for the concentration of AZO NPs used.

**TABLE 2:** Zone of inhibition (ZOI) diameter of ZnO samples against (a) *E. coli*, (b) *A. baumannii*, (c) *S. aureus*, (d) MRSA3, and (e) MRSA6 was measured from *n* = 3 and one of the representative data was shown.

**Bacteria cells**	**Zone of inhibition (mm)**			
	**(i) ZO**	**(ii) AZO**	**(iii) Antibiotics**	**(iv) DW**
(a) *E. coli* ATCC 25922	N.D.	N.D.	17^a^	N.D.
(b) *A. baumannii* ATCC 19606	N.D.	N.D.	19^a^	N.D.
(c) *S. aureus* ATCC 25923	11	14	37^b^	N.D.
(d) MRSA3	11	14	12^b^	N.D.
(e) MRSA6	11	13	N.D.	N.D.

*N.D. indicates that the zone of inhibition was not detected. Antibiotics, ^a^Polymyxin B or ^b^Kanamycin was used as a control for Gram-negative or -positive bacteria, respectively. DW, deionized water. Data shown here is one of the representative from *n* = 3.*

However, the AZO sample was effective in inhibiting the growth of Gram-positive bacterial cells including *S. aureus* (Figure 4c), MRSA3 (Figure 4d) and MRSA6 (Figure 4e). This observation corroborated the MIC determinations (Table 1). The effectiveness of the synthesized sample of AZO against the MRSA strains substantiates its potential to be used as a nanoweapon against AMR Gram-positive bacterial cells. Additionally, the MIC and ZOI data indicate that Gram-positive bacteria are better targets for AZO NPs than Gram-negative bacteria.

#### Morphological Characterization of Bacteria

**FIGURE 5:**
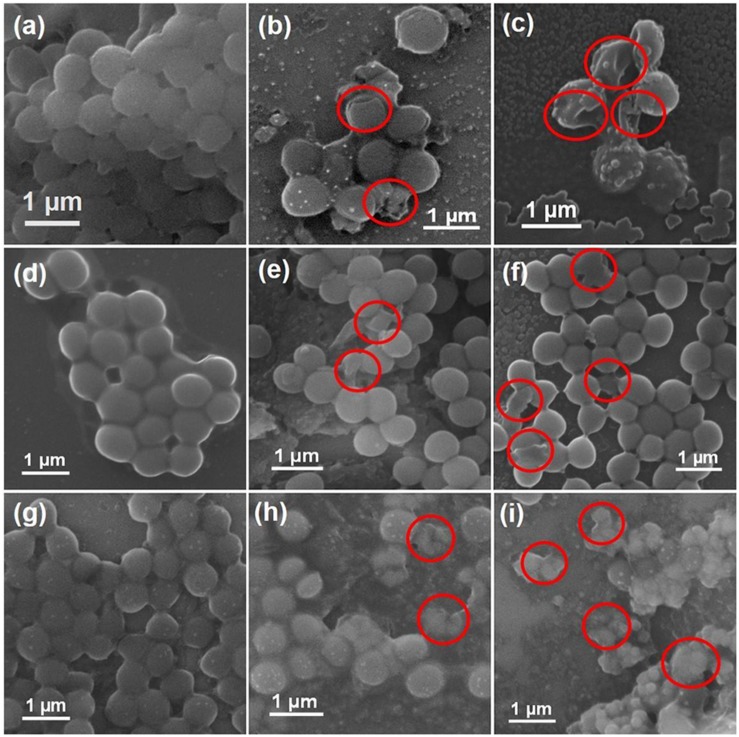
Scanning electron microscopy (SEM) images of bacterial cells. Samples of *S. aureus* **(a)** untreated and treated with **(b)** 10 μg/mL and **(c)** 20 μg/mL of AZO. Samples of MRSA3 either **(d)** untreated or treated with **(e)** 10 μg/mL and **(f)** 20 μg/mL of AZO. Samples of MRSA6 either **(g)** untreated or treated with **(h)** 10 μg/mL and **(i)** 20 μg/mL of AZO. Red circles indicate areas of cell membrane disruption.

Given the antimicrobial efficacy of AZO NPs against Gram-positive bacteria, the morphological features of standard and AMR strains of *S. aureus* (standard, MRSA3, and MRSA6) before and after exposure to AZO nanoparticles were evaluated by SEM. The SEM images of bacterial cells treated or untreated with AZO NPS is shown in Figure 5. In the untreated *S. aureus* cells, a smooth and intact surface was clearly visible (Figure 5a). On the other hand, some morphological changes such as membrane damage were seen in *S. aureus* treated with different concentrations of AZO (Figures 5b,c). Similar activity was seen in MRSA strains (MRSA3 and MRSA6) when comparing the untreated groups (Figures 5d,g), which both exhibited smooth surfaces, with the groups treated with different concentration of AZO NPs for MRSA3 (Figures 5e,f), and MRSA 6 (Figures 5h,i) which showed wrinkling and damage of the cell walls. Considerable damage was observed upon binding of the nanoparticle to the bacterial cell membrane (Figures 5e,f,h,i) to confirm the antibacterial effectiveness of AZO NPs. Therefore, the efficacy of AZO NPs against *S. aureus* and MRSA strains was successfully approved by the SEM micrographs.

It is well known that Ag and ZnO NPs are established antibacterial agents; however, very little is known about their mechanism of antibacterial activity. In this study, we explored one possible mechanism of Ag and ZnO NP antibacterial activity. It has been shown that some of the antibacterial activity of Ag and ZnO NPs may be attributed to a direct interaction between AZO NPs and the bacterial cell wall (Matai et al., 2014). The bacterial cell wall is generally negatively charged (Ghosh et al., 2012), which enables electrostatic interaction with the Zn^2+^ and Ag^+^ present in AZO NPs (which were identified in our ZO and AZO NPs by TEM analysis and XPS). Disruption of the bacterial cell membrane by Ag-ZnO NPs can be another potential mechanism for antibacterial activity (Naskar et al., 2020), which we have corroborated here using SEM. Membrane damage generally results in increased inhibition of DNA/plasmid replication by Zn^2+^/Ag^+^ ions and the production of proteins/enzymes that affect bacterial cell functioning and contribute to cell death. Moreover, membrane disruption can also cause leakage of the intracellular material, which may shrink the cell and ultimately result in cell lysis (Yasir et al., 2019).

## Conclusion

In summary, we have developed a new strategy for the one-pot synthesis of Ag-ZnO (AZO) nanoparticles using a low-temperature solution technique. The synthesized AZO sample showed admirable antibacterial activity against *S. aureus* bacteria including their AMR (MRSA) strains. Moreover, the antibacterial activity of the AZO sample was more specific toward Gram-positive bacteria than Gram-negative bacteria. This cost-effective simple synthesis strategy can be used as a platform to develop different metal oxide nanomaterials, which can be further used for targeted biomedical applications and may be useful as antibacterial agents to address the ever-increasing problem of AMR bacteria.

## Data Availability Statement

All datasets generated for this study are included in the article/supplementary material.

## Author Contributions

AN, SL, and KK contributed conception and design of the study, and wrote the manuscript. AN and SL performed the experiments. KK supervised the study. All authors contributed to the manuscript revision, and read and approved the submitted version.

## Conflict of Interest

The authors declare that the research was conducted in the absence of any commercial or financial relationships that could be construed as a potential conflict of interest.
